# Account of a Remarkable Case, in Which a Considerable Part of a Female Body Was Converted into Fatty Matter

**Published:** 1800-01

**Authors:** S. M. Mansfield

**Affiliations:** Thrapston, Northamptonshire


### For the Medical and Physical Journal.

*Account of a remarkable Cafe, in which a confiderable Part of a Female Body was converted into Fatty Matter. By* Mr. Mansfield, Surgeon, Thrapfton, Northamptonfhire.

THE curiofity of the philofophical part of mankind has been of late much excited, by the accounts which we have received, of human as well as other mufcular flefh, being converted into fat by flow putrefaction. I have now an opportunity of adding to the ftock of information on this fubject, a remarkable cafe, which I fhall defcribe, and which you will probably think deferves to be recorded as an extraordinary one; and perhaps a fingular inftance may not fo foon occur again, in which the period of time confumed in the procefs, can be fo well afcertained. The examination of it afforded me great gratification; and I have that reafon at leaft for conceiving, that the account of it may be acceptable to many others.

A young woman of the parifh of Iflix, near Thrapfton, in Northamptonfhire, feventeen years of age, of a middle ftature, and thin in her make, was drowned in the river Ren, near that place, upon the 1ft of December, 1798; and the body was not difcovered until the 27th of November, 1799, at a place about four miles off, by land; and, from the circuitous courfe of the ftream, about as far again by water.

As foon as I heard that it was found, I loft no time in going to fee it, glad of an opportunity to fatisfy myfelf, in afcertaining the truth of what had been faid upon this fubject, viz. that mufcular flefh immerfed in a ftream of water, will in time become a fatty fubftance, not fubject to farther decay in the water, nor to the ufual effects of putrefaction.

I found the fact fully proved: the parts fubject to this change, had the appearance, as it firft ftruck me, of a lump of fat. The bones of the head, neck, and ribs, were nearly cleared of all the foft parts, the cheft was quite void of its contents: the ribs themfelves as clear of flefh as poffible; but from thence downwards, all was firm and perfect. The abdomen and its contents remained in a natural ftate; as did the thighs and legs; and all had the fame external appearance with a thick covering of fat, crack'd all over like the peel of a wall-nut when ripe.

There was nothing that had any refemblance to fkin, but an upperupper ftratum about half an inch deep, which was difpofed to feparate from the mufcular parts; this I took at firft to be adipofe membrane very much thickened, as it was of a loofe and friable texture; but it is probable that this was fkin, which had undergone the fame change. Under this was a firm and white ftratum of fat, very confiderably thicker than the former; this appeared to be the upper part of the mufcles, as it was not feparated from the under part, which latter was pale, firm, and attached to the bone, as in a frefh ftate; upon cutting into this laft fubftance, however, I found it very offenfive (though the upper part was not fo) to the fmell.

Not any of this fubftance was cryftallized like fpermaceti; all the remaining part of the body had the exact appearance externally of a boiled ham, when ftripped of the fkin, both as to colour and fubftance.

That the putrefaction in this cafe was flow, is eafily accounted for, as the body was in the water all the preceding winter, which was a very cold one,—the fummer too was uncommonly cold and rainy; and on this latter account, the ftream unufually rapid. From fome accidental caufe, the body had been detained all the time under the furface of the water, perhaps by the clothes having been entangled by the roots of a tree.

That there could be no doubt of this being the fame body that was loft at the above period; it may be proper to obferve, that the fhoes and buckles remained on her feet, which the parents immediately identified.

It may be difficult to determine, at what precife period of time the change began to take place, and how long it would have been, before the remainder of the mufcular flefh would have undergone the fame alteration; but it is clear, that the procefs of converting a confiderable part into fatty matter of a combuftible nature, may be compleated in the period of twelve months; which may fuggeft a hint as to the propriety of making this change an article of manufacture.

I have been much gratified by this inveftigation, and in having it in my power to give an account of it to thofe, whofe curiofity, like mine, may have been excited by this extraordinary difcovery, refpecting the change taking place in animal matter, from flow putrefaction.

*Thrapfton, Northamptonfhire,*

*Dec.* 7, 1799.

S. M. MANSFIELD.

