# Errata

**Published:** 1800-03

**Authors:** 


### ERRATA.

Vol. III. page 10, line 13, for *singular*, read *similar*, and dele *so*.

line 18, for *Islix*, read *Islip*.

line 20, for *Ren*, read *Nen*.

